# Structural equation model was used to evaluate the effects of soil chemical environment, fertility and enzyme activity on eucalyptus biomass

**DOI:** 10.1098/rsos.221570

**Published:** 2023-04-12

**Authors:** Jian Tang, Junyu Zhao, Zuoyu Qin, Lijun Chen, Xianchong Song, Qin Ke, Lichao Wu, Yuanyuan Shi

**Affiliations:** ^1^ Guangxi Research and Development Center for New Forestry Fertilizer, Key Laboratory of Central South Fast-growing Timber Cultivation of Forestry Ministry of China Nanning, Guangxi Zhuang Autonomous Region Forestry Research Institute, Nanning 530002, People's Republic of China; ^2^ Key Laboratory of Cultivation and Protection for Non-Wood Forest Trees of National Ministry of Education, Central South University of Forestry and Technology, Changsha 410004, People's Republic of China

**Keywords:** Fourier transform infrared, soil properties, organic functional groups, southern subtropics, soil structure evolution

## Abstract

This paper aims to reveal the effects of multi-generational succession of eucalyptus on soil fertility, organic structure and biological properties. Soil samples were collected from eucalyptus plantations of different stand ages (5, 11, 17 and 21 years old) in a typical area in south Asia, soil organic fraction structure and content characteristics were investigated using Fourier transform infrared (FTIR), and structural equation modelling (SEM) was used to explore influences of soil fertility, enzyme activity and organic fraction on stand biomass. FTIR analysis showed that 11 infrared absorption peaks existed in the soils of this study area, attributed to silicates, aromatics, carbonate ions, sugars, esters, polysaccharides, aliphatic hydrocarbons and phenolic alcohols. Combined with the results of peak area integration, the content of esters, aromatics and phenolic alcohols was significantly higher in 17- and 21-year-old stand soils than in control soils. The results of SEM showed that organic components were negatively related (*p* < 0.01) to enzyme activity and biomass, with standardized coefficients of 0.53 and 0.49, respectively. In summary, multi-generation succession of eucalyptus trees can change the structure of soil organic functional group composition and promote the enrichment of aromatic and phenolic alcohol functional groups. Such changes can directly inhibit the increase in eucalyptus biomass and indirectly negatively affect biomass by inhibiting enzyme activity.

## 1. Introduction

Eucalyptus, one of the world's three fastest-growing tree species, is widely planted in tropical and subtropical regions and plays an important role in regional economic development and global timber supply [1,2]. However, in pursuit of higher economic benefits, the crop rotation cycle of eucalyptus has been gradually shortened from 7–8 years to 4–5 years, and problems such as soil consolidation, nutrient imbalance and reduced accumulation caused by the high-intensity continuous planting pattern have been reported [3]. With the rapid growth of eucalyptus plantations in southern China, Chinese society is increasingly concerned about the ecological problems caused by eucalyptus plantations [4]. Existing studies have shown that successive multi-generation planting of eucalyptus causes shortages of soil nitrogen (N), phosphorus (P) and potassium (K), as well as a decrease in enzyme activity [5], and the growth of the plants themselves is also suppressed [6,7], but the intrinsic drivers that cause these changes are not clear. The growth of successive planting eucalyptus plantations can be maintained to some extent by increasing the amount of fertilizer inputs, but this measure is not sustainable [8]. More important is the long-term impact, how has the soil organic environment changed after multiple generations of eucalyptus succession? What is the impact of these changes on eucalyptus biomass and how can we characterize this impact? To address these questions, we characterized the soil organic fraction by infrared spectroscopy and modelled using structural equations to investigate how soil properties might change, thus predicting the direct and/or indirect causes of biomass decline after multi-generational succession of eucalyptus plantations. This work was done to provide basic theoretical support for addressing the ecological risks associated with multi-generational succession of eucalyptus plantations.

Soil health can be measured using soil fertility, organic fraction and enzyme activity, factors that interact with each other and simultaneously impact plant growth [9,10]. Studies have confirmed that successive multi-generational planting of eucalyptus affects soil fertility, enzyme activity and chemical composition [11]. Fertility is a fundamental property of soils and is closely related to plant growth and development. Soil enzyme activity is extremely important for the biochemical reactions of carbon, N and P, and significantly affects the nutrient content of eucalyptus-stand soils [12]. Continuous multi-generational planting of eucalyptus changes the size of the nutrient pool and the enzymatic activity of the soil, including changes in total N, P, K, urease and pH, and these changes further drive the evolution of the soil chemical composition and microbial community [13]. Root secretion [14] and apoplastic decomposition [15] are important ways in which plants influence the soil chemical environment. Research has suggested that soil degradation and the decline in stand biomass after successive plantings of eucalyptus are due to chemosensitization [16]. Eucalyptus root secretions contain 29 organic components, including aromatic acid esters and N-containing naphthalene heterocycles [17,18], with evidence from indoor experiments suggesting they inhibit weed growth and root activity [19]. However, other factors such as soil resilience and concentration of chemosensitive substances can alter the environmental effects of chemosensitive substances, as seen in recent field experiments [18]. Research has also confirmed that the decomposition of eucalyptus apoplastic residues produces large amounts of phenolic acids, which have an inhibitory effect on the supply of effective plant N [20]. Given this effect, the inhibitory effect of apoplastic residues on plant growth needs to be evaluated comprehensively because the content and type of phenolic acids are influenced by soil redox conditions, fertility level and pH [21].

Due to the large structural variability of organic fractions in soils, as well as the complexity of organic chemical reactions and many side reactions [22], existing studies have attempted to explain the environmental effects of chemosensitive substances by detecting the chemical fractions and contents of apoplastic leachate [23] or root secretions [24,25]. This approach is somewhat one-sided. We believe that a systematic approach should be adopted to evaluate and establish the interaction pathways between soil fertility, organic fractions and enzyme activities, and to evaluate the effects of these factors on plant growth in a comprehensive manner. Fourier transform infrared (FTIR) spectroscopy can determine the quantity and chemical structural composition by measuring infrared radiation (400–4000 cm^−1^) of organic functional groups in soils [26]. In this study, we characterized the organic chemical structure of eucalyptus plantation soils of different successive planting generations using FTIR [27]. Structural equation modelling (SEM) is a statistical technique for identifying causal relationships between different factors, extracting common features from variables to generate common factors and assessing the relationships between variables that affect each other [28]. Fertility, enzyme activity and soil organic fraction have a complex influence on each other, while they collectively influence plant growth, so it is advantageous to build causal networks to estimate the effect of each factor on growth [29]. The use of FTIR to characterize organic chemical fractions and investigate the pathways of direct or indirect effects of soil fertility, enzyme activity and organic fractions on biomass through structural equation modelling is exploratory in nature because few studies have incorporated organic chemical fractions into the pathways of effects between soil fertility and enzyme activity in eucalyptus plantation forests in southern China.

In this research, we posed the following hypotheses: (i) FTIR can be used to characterize the organic chemical composition and structure of soils in multi-generation succession planted eucalyptus plantations and find differences; and (ii) it is possible that changes in soil chemical structure inhibit soil enzyme activity and reduce the effectiveness of soil fertility, which in turn leads to a decrease in plant growth. The objectives of this study were as follows: (i) to determine soil fertility and enzyme activity indicators, and to characterize the structural characteristics of soil organic fractions using FTIR; (ii) to use structural equation modelling to establish the interactions between soil fertility, enzyme activity and chemical composition and the pathways of effects on plant biomass; and (iii) to investigate the pathways of effects of continuous multi-generational planting of eucalyptus on direct or indirect causes of biomass decline.

## 2. Material and methods

### 2.1. Overview of the study area

The test site was set up in Guangxi state-owned Dongmen forestry field (21°35′–22°41′ N, 107°72′–109°56′ E). The subfield is located in the subtropical monsoon climate zone, with more than 1800 h of sunshine, 105–110 kcal cm^−2^ yr^−1^ of solar radiation, high temperature, abundant heat and abundant rainfall. The average annual temperature is 21–22°C, extreme high temperature 38.4°C, extreme low temperature −2.6°C observed in recent years, greater than or equal to 10°C of the active cumulative temperature of 7500°C. The annual rainfall is 1200–1300 mm, the annual evaporation is 1600–1800 mm, and the relative humidity is approximately 79%. According to the Chinese Soil Occurrence Classification, the soil type in the study area is Allitic-Udic ferrosols, according to world reference basic of soil resources, the soil type is ferric acid soil, which developed from the Quaternary laterite parent material. Eucalyptus plantations in the study area were operated in the same way, with manual clearing of weeds and shrubs followed by spraying of herbicides (10% glyphosate agent) on the stumps, planting of histone seedlings in 20 cm deep soil pits, application of 0.25 kg of basal fertilizer after planting, and stand planting density of 1.5 × 4 m with 1668 trees per hectare, followed by 0.5 kg of follow-up fertilizer (N : P : K of 15 : 6 : 9) in June each year.

### 2.2. Experimental design and soil sample collection

Sample plots were laid out and soil samples were collected on 3 June 2021. Four different stands of 5, 11, 17 and 21 years old (abbreviated as T0, T1, T2 and T3, respectively), were selected and harvested every 5 years. The stumps were retained for re-sprouting and renewal, and the best growing one was selected and retained three to six months after sprouting. Eucalyptus stands of 10, 14 and 19 years old were harvested once, twice and three times, respectively. These trees were 5 years old at the time the experiment was conducted. All of the eucalyptus plantations were preceded by native evergreen broadleaf forests, except for T0 where the preceding species was *Litchi chinensis* Sonn.

The experiment was conducted in a randomized group design with 20 standard sample squares (20 × 20 m) in four stand types, each containing five replicates. Thirty live trees were randomly selected in the sample squares, tree height and diameter at breast height (DBH) were measured, and the average value was calculated. Five live trees closest to the average tree height and DBH were selected, and inter-root soil (soil attached to the root system was defined as inter-root soil) was collected. The soil samples were mixed well and collected in quadrats with 500 g of soil samples, marked and put into ice boxes (0°C), and brought back to the laboratory for testing (table 1).

**Table 1.** Sample plot survey.

block	longitude	latitude	altitude (m)	slope (°)	aspect	height (m)	DBH (cm)
T0	108°8′10″ E	22°40′17″ N	188	26	northeast	24.8	14.5
T1	108°6′12″ E	22°39′59″ N	192	24	north	25.3	16.3
T2	108°12′17″ E	22°41′46″ N	196	27	north	23.8	15.1
T3	108°11′49″ E	22°42′0″ N	203	24	northeast	21.9	16.3

### 2.3. Soil property determination

Soil pH was determined using a pH meter in the supernatant at a 5 : 1 water to soil ratio [30]. Soil organic matter (SOM) was determined using the potassium dichromate oxidation with an external heating method. Soil total N and alkaline N were determined by semi-automatic Kjeldahl N tester [31]. Total and effective phosphorus were determined by digestion method [32] and Mehlich 3 liquid leaching method [33], respectively, using a fully automated interrupted chemical analyser. Total potassium (TK) and available potassium (AK) were measured with digestion and Mehlich 3 methods and a flame photometric detector, respectively [34]. Soil sucrase (SC) and amylase were determined by 3, 5-dinitrosalicylic acid colorimetric method; cellulase-nitro salicylic acid colorimetric method (72 h glucose mg (10 g)^−1^; urease (URE) was determined by sodium phenol-sodium hypochlorite colorimetric method; acid phosphatase (ACP) activity was determined by sodium phenyl phosphate colorimetric method [35].

### 2.4. Soil infrared spectral data acquisition

The soil samples were removed from the fresh bags, fully air dried, ground and passed through a 0.149 mm soil sieve. The sieved naturally air-dried soil samples were mixed with dried potassium bromide (spectrally pure) at a mass ratio of 1 : 90, ground manually with an agate mortar and pestle, compacted by a tablet press at 15 kPa, and placed in a Thermo Nicolet iS50 FTIR spectrometer (ThermoFisher Scientific, USA) to be measured. With KBr as the background value, the spectra were collected in the range of 400–4000 cm^−1^, with a resolution of 4 cm^−1^, and the scanning frequency was 64 times. Each sample was repeated five times, and the average value was taken after similarity comparison; the sample IR spectra were obtained after deducting the background value. The raw spectral data were corrected using the calibration function that comes with OMNIC to remove the influence of baseline, and then standard normalization was performed to remove the difference in weighing between different samples.

### 2.5. Structural equation modelling

Soil fertility, enzyme activity and organic fraction data were analysed using IBM SPSS Statistics (v. 24.0) software (IBM, Armonk, NY, USA). Data were analysed using one-way ANOVA with Duncan's test for comparison of means, factorial at five loci and structural equation modelling using the software package AMOS (Arbuckle [36]).

### 2.6. Data analysis and processing

The analysis of basic infrared spectra, peak finding and standardization were completed by OMNIC Specta, Origin 2018 was used to integrate the area of each functional group characteristic peak and calculate the average value and percentage, and SPSS 19.0 was used for basic data analysis and graphing.

## 3. Results

### 3.1. Soil fertility index

Among the chemical indicators measured, all of them differed significantly (*p* < 0.05) in different generations of eucalyptus plantation soils, except for the total K content. Soil pH (3.56) was significantly lower (*p* < 0.05) in T3 soil of high-generation eucalyptus plantations than in the rest of the stands. SOM content of T2 and T3 soils was 34.39 and 36.17 g kg^−1^ significantly higher (*p* < 0.05) than that of the lower generation (T0 and T1) eucalyptus plantations, respectively. The highest total N content was found in stand T3, which was significantly higher by 67.88% compared with T0 (*p* < 0.05). The maximum value of total P content was found in the sprouting second-generation stand treatment (T2) with 2.73 g kg^−1^. Of fast-acting nutrients, AN, AP and AK contents in the T1 treatment were 219.8, 10.8 and 61.0 mg kg^−1^, respectively, which were significantly higher than those in the remaining generations of eucalyptus plantations (*p* < 0.05), while fast-acting nutrients were generally lower in the soils of high-generation plantations T2 and T3 (table 2).

**Table 2.** Soil chemical properties. Different lowercase letters in the same column indicate significant differences between indicators (*p* < 0.05).

block	pH	OM g kg^−1^	TN g kg^−1^	TP g kg^−1^	TK g kg^−1^	AN mg kg^−1^	AP mg kg^−1^	AK mg kg^−1^
T0	3.94 ± 0.18^a^	24.36 ± 11.27^b^	2.18 ± 0.17^c^	2.01 ± 0.14^b^	9.32 ± 1.18^a^	179.9 ± 23.8^b^	8.6 ± 0.87^a^	46.36 ± 8.51^b^
T1	3.75 ± 0.21^b^	26.03 ± 16.95^b^	2.89 ± 0.27^b^	1.52 ± 0.21^c^	7.75 ± 1.85^a^	219.8 ± 27.6^a^	10.8 ± 0.95^a^	61 ± 15.21^a^
T2	3.71 ± 0.11^b^	34.39 ± 13.14^a^	3.17 ± 0.11^b^	2.73 ± 0.17^a^	7.34 ± 1.54^a^	159.3 ± 26.1^c^	4.06 ± 1.05^b^	41.23 ± 3.96^c^
T3	3.56 ± 0.08^c^	36.17 ± 4.88^a^	3.66 ± 0.16^a^	0.78 ± 0.11^d^	8.28 ± 1.34^a^	157.0 ± 18.9^c^	4.46 ± 0.41^b^	56.23 ± 26.1^b^

### 3.2. Soil enzyme activity

Among the measured enzyme activities, except cellulase, all enzyme activity indicators differed significantly (*p* < 0.05) among generations of eucalyptus plantation soils. Sucrase, β-glucomannase, urease and ACP were significantly lower in the T3 treatment than in the rest of the stand types (*p* < 0.05), indicating that planting time mainly affected the activities of the above enzymes. There was no significant difference in cellulase activity among generations, indicating that this enzyme was not significantly affected by planting time (*p* > 0.05). Amylase activity showed significantly lower (*p* > 0.05) in low-generation plantations T0 (8.66 mg (g 24 h)^−1^) (table 3).

**Table 3.** Soil enzyme activities. Different lowercase letters in the same column indicate significant differences between indicators (*p* < 0.05).

block	SC mg (g 24 h)^−1^	AMY mg (g 24 h)^−1^	CEL mg (g 72 h)^−1^	NAG mg (g 24 h)^−1^	URE mg (g 24 h)^−1^	ACP mg (g 24 h)^−1^
T0	6.18 ± 0.57^a^	8.66 ± 3.87^b^	0.30 ± 0.07^a^	1.03 ± 0.07^a^	0.25 ± 0.11^a^	2.09 ± 0.21^a^
T1	7.53 ± 0.88^a^	10.45 ± 4.77^a^	0.34 ± 0.13^a^	1.19 ± 0.17^a^	0.27 ± 0.04^a^	2.44 ± 0.54^a^
T2	5.30 ± 0.74^b^	12.18 ± 3.65^a^	0.30 ± 0.07^a^	1.14 ± 0.32^a^	0.17 ± 0.02^a^	1.45 ± 0.35^b^
T3	4.38 ± 0.18^c^	11.96 ± 2.87^a^	0.35 ± 0.17^a^	0.62 ± 0.18^b^	0.13 ± 0.05^b^	1.87 ± 0.27^b^

### 3.3. Characteristics of soil organic fraction

**Figure 1.**
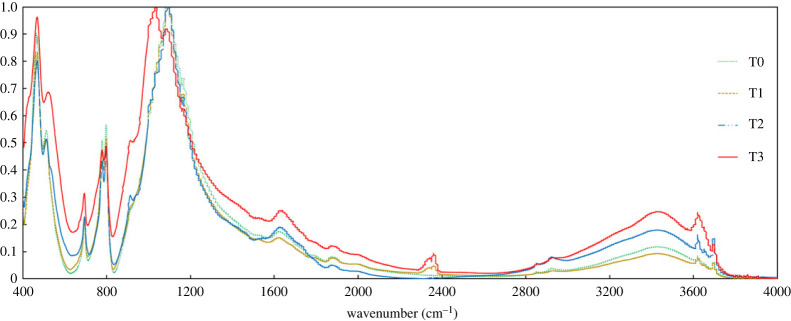
Infrared spectrum characteristics of soil of eucalyptus plantations of different generations.

The infrared spectrogram (figure 1) shows that the infrared absorption spectral characteristics of eucalyptus plantation soils with different planting years are basically the same, and the overall performance is the absorption characteristics of silicate (similarity 75.31%). The differences are mainly reflected in the infrared spectral absorption intensity, in which a total of 11 absorption peaks of functional groups appear, and the wavenumbers are 469, 694, 778, 800, 1088, 1165, 1631, 2361, 2929, 3423, 3620 cm^−1^, through the spectral analysis, characteristic peaks, absorption intensity and other spectral index characteristics (Alvarez-Ordóñez & Prieto [37]; Margenot *et al*. [38]; Prommer *et al*. [39]), the wavenumber functional group designations were assigned to silicates, aromatics, carbonate ions, sugars, esters and polysaccharides, aliphatic hydrocarbons and phenolic alcohols. The intensity of the infrared spectral absorption peaks increased with the growth time. Compared with T0 and T1, new absorption peaks of esters and polysaccharides appeared at 2361 cm^−1^ in the T2 and T3 eucalyptus plantations. Infrared spectra of T3 treatment soils showed significantly higher absorption peaks at 694, 1631, 2361, 3423 and 3620 cm^−1^ than the rest of the generations of eucalyptus plantation soils (*p* < 0.05), and these regions mainly characterized aromatic bonds (e.g. C–H, C=C, C=O). The absorption peaks of T3 at 1088 and 1165 cm^−1^ showed a more pronounced red shift towards lower wavenumbers, representing a decrease in the energy required for vibration.

### 3.4. Soil infrared spectrum absorption peak area integration

There were significant differences in the peak area integrals of the main peaks of the infrared spectra of eucalyptus plantation soils with different planting years. The bands with larger peak areas were mainly found at 1088, 1165, 1631 and 3423 cm^−1^, with a range of 86.59% to 90.18% in the different treatments, corresponding to functional groups of esters, aromatics and phenolic alcohols. The relative peak area integrals of the IR spectra were significantly different at 694, 1088, 2361, 2929, 3423 and 3620 cm^−1^ (*p* < 0.05). The peak area integrals of the T2 and T3 treatments were significantly higher than those of the T0 and T1 treatments at 2929, 3423 and 3620 cm^−1^. New absorption peaks appeared at 2361 cm^−1^ in the T2 and T3 treatments. Meanwhile, the peak area integral of the T3 treatment at 2361 cm^−1^ was significantly increased by 78.37% compared with that of the T2 treatment, and this band mainly characterized the absorption peaks of functional groups of aromatic substances (table 4).

**Table 4.** Integral of relative peak area of main peaks in soil infrared spectra. Different letters indicate significant differences between treatments (*p* < 0.05, *N* = 5).

wavenumber (cm^−1^)	block			
	T0	T1	T2	T3
469	8.19 ± 0.18^a^	7.88 ± 0.16^a^	8.753 ± 0.23^a^	8.21 ± 1.02^a^
694	0.61 ± 0.08^d^	0.74 ± 0.06^cd^	1.03 ± 0.58^bc^	2.03 ± 0.59^a^
778	1.22 ± 0.28^a^	1.08 ± 0.16^a^	1.15 ± 0.41^a^	1.55 ± 0.32^a^
800	1.72 ± 0.34^a^	1.81 ± 0.39^a^	1.68 ± 0.33^a^	2.12 ± 0.31^a^
1088	11.15 ± 2.04^c^	11.41 ± 0.89^c^	13.24 ± 0.63^b^	16.01 ± 0.85^a^
1165	17.54 ± 1.13^a^	16.28 ± 2.16^a^	15.98 ± 2.18^a^	17.87 ± 1.96^a^
1361	4.459 ± 0.82^b^	3.951 ± 0.71^c^	4.436 ± 1.12^b^	6.567 ± 0.63^a^
2361	0.18 ± 0.07^c^	0.07 ± 0.05^c^	0.37 ± 0.11^b^	0.66 ± 0.03^a^
2929	0.50 ± 0.22^b^	0.43 ± 0.31^b^	1.10 ± 0.36^a^	1.17 ± 0.17^a^
3423	3.48 ± 0.98^b^	2.73 ± 0.06^b^	5.46 ± 0.65^a^	7.4 ± 1.82^a^
3620	0.64 ± 0.41^b^	0.58 ± 0.12^b^	1.28 ± 0.09^a^	1.94 ± 0.84^a^

### 3.5. Structural equation model

**Figure 2.**
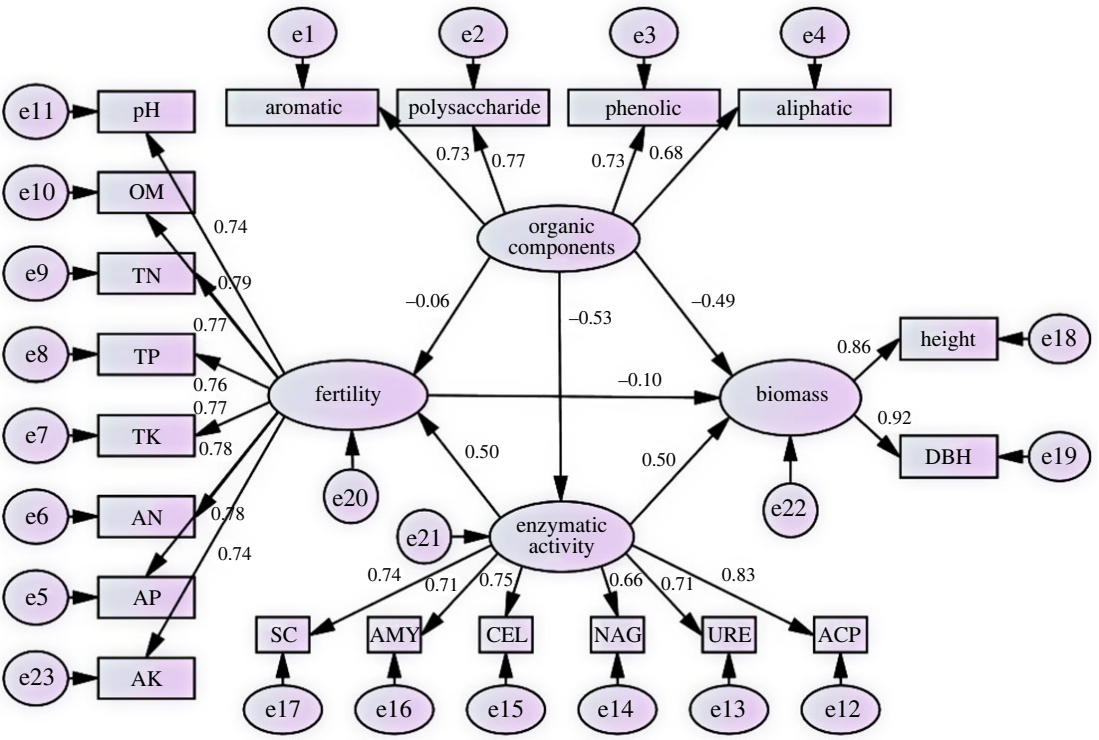
Structural equation model of organic components, fertility, enzyme activity and biomass. pH, pH value; OM, organic matter; TN, total nitrogen; TP, total phosphorus; TK, total potassium; AN, available nitrogen; AP, available phosphorus; AK, available potassium; SC, sucrase; AMY, amylase; CEL, cellulase; NAG: glucosidase; ACP: acid phosphatase; URE: urease.

Under the management measures of multi-generational succession, indicating that among the three latent variables, organic components had a negative effect on fertility and enzyme activity, while enzyme activity had a significant positive effect on both fertility and biomass. The model fit results showed that the model CMIN/DF = 1.139, RMSEA were less than 0.08, and the model fit was good. The loadings of the apparent variables could explain the main influencing factors of each latent variable. In soil fertility, the differences in loadings of the apparent variables were small, the largest of which was ACP (0.83) and the smallest was NAG (0.66) in enzyme activity. There were four significant paths of influence (*p* < 0.01): organic components had a significant negative influence (*p* < 0.01) on the enzymatic activity and biomass fertility. Enzymatic activity had a significant positive effect on fertility and biomass fertility (*p* < 0.01) with standardized coefficients of 0.50 and 0.50, respectively, but the pathway of organic components affecting fertility (−0.06) and thus biomass (−0.10) was not significant (*p* > 0.05) (figure 2).

According to principal component analysis, analysis of variance and structural equation modelling, the increase in soil organic fractions such as aromatic, polysaccharide, phenolic and aliphatic, either directly or indirectly negatively affected fertility and enzyme activity under multi-generational succession conditions, with the main influencing factors being polysaccharides and phenolic substances. The difference in the load values of each significant variable for soil fertility was small, and the direct effect of fertility on biomass was also small. Thus, the reduction of biomass after continuous planting was probably due to the indirect effect of soil organic components through the inhibition of enzyme activity.

## 4. Discussion

### 4.1. Changes in soil fertility and biological property

We found that the management measures of multi-generational succession had a significant negative effect on soil pH, available nutrient elements and enzyme activities (SC, NAG, URE and ACP) in eucalyptus plantations in the study area. These same management measures had a positive effect on the accumulation of organic matter and total nitrogen content, indicating that the management measures of multi-generational succession did not have a completely negative effect on soil fertility. Consistent with the findings of previous studies [40], soils showed a trend of acidification after multi-generational succession, but soil OM and TN were significantly elevated in sprouted second-generation stands, but fast-acting nutrient elements generally showed a decreasing trend. The soil was exposed due to violent soil disturbance (slash and burn) during the new plantation process, which destroyed the soil structure and caused a large loss of SOM and nutrient elements in the T0 and T1 stands [41,42]. After mature felling of newly planted forests, the sprouting and re-growth operation was adopted, which does not require clearing of harvesting residues, so nutrients were gradually accumulated within the soils of second- and third-generation stands [43], while the accumulation of nutrient elements is also promoted by heavy fertilization. It has been shown that long-term management practices such as inorganic fertilizer application and manual weeding can lead to negative impacts such as soil acidification, increased bulk weight and decreased soil fertility quality [44]. Soil acidification accelerates the fixation of free phosphorus elements in the soil [42], and soil compaction reduces the rate of carbon and nitrogen cycling in the stand [45], thus exhibiting a significant reduction in fast-acting nutrients in high-generation plantation soils. Studies have shown that soil enzyme activity is significantly associated with stand nutrient cycling and is involved in the mineralization process of organic matter [35]. Continuous planting significantly affected sucrase, urease and ACP activities, and the decrease in the activities of these three enzymes indicated that the cycling of carbon, nitrogen and phosphorus in the soil was blocked [46]. Thus, the quick-acting nutrients in the soil of high-generation eucalyptus plantations were significantly reduced.

### 4.2. Changes in soil organic components

We characterized the soil organic fraction by FTIR and found that the functional groups of organic fraction in the soil also tend to be homogeneous after multiple generations of succession, which reduces the effectiveness of organic carbon, indicating that multiple generations of succession are not conducive to the efficient cycling of organic carbon in eucalyptus plantation soils (3620 cm^−1^), which are more stable carbon sources and difficult to be used by soil microorganisms, thus affecting the nutrient conversion rate [47]. Meanwhile, new unsaturated (C=C, C=O, 2361 cm^−1^) production was found in the high-generation T3 soils, indicating that successive plantings change their native soil organic fraction and structure [48,49]. The results of least significance difference test (LSD) multiple comparisons showed that with the increase of successive planting generations, the peak area integrals at 2929, 3423 and 3620 cm^−1^ were significantly higher in the T2 and T3 treatments than in the T0 and T1 (*p* < 0.05), and the corresponding functional groups were attributed to aliphatic and phenolic alcohols [50]. The increase in the content of these functional groups was significantly correlated with plant root uptake and metabolism [51], indicating that the interaction between plant roots and soil remained active after the multi-generational succession [52]. These substances gradually increased in the soil organic fraction after multi-generational successions, showing a certain degree of enrichment characteristics. The results of the principal component four-quadrant plots showed that aliphatic hydrocarbons, phenolic alcohols and aromatics (2929, 3423, 1631, 3620 and 469 cm^−1^) gradually dominated the soil organic fraction as the successive planting generations increased. The results of the principal component analysis of the FTIR functional group absorption peaks in plantation soils of different generations showed that the T0 sample sites were distributed in the second, third and fourth quadrants in newly planted forests, while the T3 sample sites were all distributed in the first quadrant, and the distribution of sample sites was more concentrated with the increase of successive plantation generations. Also, the T3 main effective functional groups (1631 and 3620 cm^−1^) belonged to aromatic and phenolic alcohols. One study showed that these substances are toxic and have a certain inhibitory effect on the growth of shrubs and weeds [1]. Thus, the enrichment of these substances in older-stand soils may cause an increase in growth inhibition by self-toxicity.

In this study, the structural characteristics of soil organic functional groups were characterized by infrared spectroscopy, and it was found that with the increase of successive planting generations, the homogeneity of plantation community characteristics [53] caused the main functional groups in their organic fractions to become homogeneous. This homogeneity was linked with improving soil microbial diversity and activity to promote the decomposition of complex organic compounds such as aliphatic hydrocarbons, phenols and alcohols, and aromatics, which may be an effective way to solve the obstacles to successive planting of eucalyptus.

### 4.3. Effects of the successive planting of eucalyptus on soil characteristics

We explored the pathways of the formation of succession barriers in eucalyptus through structural equation modelling and found that changes in organic components had direct or indirect inhibitory effects on biomass. The increase in aromatic, polysaccharide and phenolic-like functional groups may directly inhibit eucalyptus biomass and indirectly affect eucalyptus growth through inhibition of enzyme activities. Structural equation modelling analysis showed that the coefficient of effect of fertility on biomass (−0.1) was non-significant, and we could not attribute the formation of successional planting barriers to changes in measured soil fertility, although plants obtain nutrient elements directly from the soil [54]. Soil fertility did not show significant degradation after multiple generations of successive planting due to long-term heavy application of chemical fertilizers [49,55]. All three latent variables had direct effects on biomass to varying degrees, with path coefficients for organic fraction (−0.49), enzyme activity (0.50) and fertility (−0.10), where the effects of organic fraction and enzyme activity were significant. In agreement with our hypothesis, the evolution of soil organic fraction content and structure could be the causal factor for the generation of succession barriers. The organic fractions with high loading values were aromatic (0.73), polysaccharide (0.77) and phenolic (0.73), and the enrichment of this group may be related to root secretions, or to direct interactions with root symbiotic and root-associated microorganisms [11,56]. At the same time, as the higher carbon source utilization by soil microorganisms is in the carbohydrate group [57], an increase in aromatic substances may reduce the efficiency of the carbon cycle, and this change may not be conducive to an increase in the capacity of the soil carbon pool from the point of view of plant growth and carbon cycling. The focus of this study was on the effect of soil chemistry on plant growth and the lack of consideration of microbial community structure. Soil microorganisms are more sensitive to changes in carbon sources [58], and it is worthwhile to further investigate whether changes in soil organic chemistry composition have an impact on microbial community structure.

## 5. Conclusion

We explored the use of FTIR to characterize soil organic fractions of eucalyptus plantations of different generations and evaluated the interaction between soil fertility, enzyme activity and organic fractions. In addition, we made predictions on the effect of the above factors together on the biomass of eucalyptus plantations through structural equation modelling. With the increase of continuous planting time, the soil of eucalyptus plantations gradually acidifies and the content of fast-acting nutrients decreases. Despite these changes, the soil total nitrogen and organic matter content had a significant increase compared with the new plantation due to the heavy fertilization and retention of harvesting residue management measures. The results of our structural equation modelling suggest that the organic fraction and enzyme activity had significant effects on eucalyptus biomass, while the path of fertility effects on biomass was not significant. The organic fraction might have directly inhibited the biomass of eucalyptus, and indirectly negatively affected the biomass by inhibiting the soil enzyme activity. Enzyme activity was predicted to have a direct positive effect on both soil fertility and biomass, but fertility contributed less to the accumulation of biomass. Considering the loading factors of significant variables in the organic fraction, we suggest that the enrichment of aromatic, polysaccharide and phenolic substances may be the main cause of biomass decline in high-generation eucalyptus plantations.

## Data Availability

Raw data storage of soil and Fourier transform infrared spectroscopy (http://10.57760/sciencedb.j00075.00005).
